# Urinary Excretion of Niacin Metabolites in Humans After Coffee Consumption

**DOI:** 10.1002/mnfr.201700735

**Published:** 2018-03-23

**Authors:** Jonathan Isaak Kremer, Katharina Gömpel, Tamara Bakuradze, Gerhard Eisenbrand, Elke Richling

**Affiliations:** ^1^ Department of Chemistry Division of Food Chemistry and Toxicology Molecular Nutrition Technische Universität Kaiserslautern Kaiserslautern Germany

**Keywords:** coffee, metabolism, niacin, nicotinamide, stable isotope dilution analysis (SIVA)

## Abstract

**Scope:**

Coffee is a major natural source of niacin in the human diet, as it is formed during coffee roasting from the alkaloid trigonelline. The intention of our study was to monitor the urinary excretion of niacin metabolites after coffee consumption under controlled diet.

**Methods and results:**

We performed a 4‐day human intervention study on the excretion of major niacin metabolites in the urine of volunteers after ingestion of 500 mL regular coffee containing 34.8 μmol nicotinic acid (NA) and 0.58 μmol nicotinamide (NAM). In addition to NA and NAM, the metabolites *N* ^1^‐methylnicotinamide (NMNAM), *N* ^1^‐methyl‐2‐pyridone‐5‐carboxamide (2‐Py), and nicotinuric acid (NUA) were identified and quantified in the collected urine samples by stable isotope dilution analysis (SIVA) using HPLC‐ESI‐MS/MS. Rapid urinary excretion was observed for the main metabolites (NA, NAM, NMNAM, and 2‐Py), with *t*_max_ values within the first hour after ingestion. NUA appeared in traces even more rapidly. In sum, 972 nmol h^−1^ of NA, NAM, NMNAM, and 2‐Py were excreted within 12 h after coffee consumption, corresponding to 6% of the ingested NA and NAM.

**Conclusion:**

The results indicate regular coffee consumption to be a source of niacin in human diet.

## 1 Introduction

Nicotinic acid (NA) and nicotinamide (NAM), also known as niacin, belong to the group of B‐vitamins (vitamin B_3_). The recommended daily intake of niacin is about 15 mg d^−1^.^1^ The average daily intake of niacin in Germany was estimated to be 36 mg for men and 27 mg for women.^2^ NA and NAM are components of the enzyme cofactors nicotinamide adenine dinucleotide and nicotinamide adenine dinucleotide phosphate, which play important physiological roles, for example, in various redox processes.^3^, ^4^ Niacin is classified as a semi‐essential vitamin due to the endogenous formation from the amino acid tryptophan in the human body, with approximately 60 mg of tryptophan being equivalent to 1 mg NA.^5^ Many foods contain niacin and niacin equivalents (NE) including meat and meat products, as well as legumes.^6^ NA is synthesized by plants, while NAM is the predominant form in animal‐based food.^7^, ^8^ The alkaloid trigonelline is degraded during coffee roasting, forming NA in amounts of 4–6% of the initial trigonelline content by demethylation. Substantial levels of NA are formed by increasing time and temperature of coffee roasting.^9^, ^10^, ^11^

After ingestion, NAM is methylated in the liver, to *N* ^1^‐methylnicotinamide (NMNAM) by nicotinamide *N‐*methyltransferase and then oxidized to *N* ^1^‐methyl‐2‐pyridone‐5‐carboxamide (2‐Py) and *N* ^1^‐4‐pyridone‐5‐carboxamide.^12^, ^13^ The major urinary metabolites of niacin are NMNAM and 2‐Py, accounting for 20–35% and 45–60%, respectively, of all niacin metabolites.^14^, ^15^ The European Food Safety Authority (EFSA) has recommended the determination of urinary metabolites, namely NMNAM and 2‐Py, as a way of measuring an individual's niacin intake.^1^ In a human intervention study performed by Lang et al. (2010), urinary levels of NA were found to increase 2 h after consuming 350 mL coffee.^16^ However, the metabolite nicotinuric acid (NUA) was only detected in urine samples from participants given pharmacological doses (up to 3 g).^14^

The chromatographic separation of niacin and its metabolites is possible by reversed‐phase HPLC,^9^, ^17^ hydrophilic liquid interaction chromatography,^16^ normal‐phase HPLC,^18^ and supercritical fluid chromatography.^19^ Detection is achieved by UV absorption,^17^ MS,^20^ or MS/MS.^9^, ^16^, ^18^, ^21^, ^22^

Most human intervention studies were conducted with the administration of pharmacological doses.^23^, ^24^, ^25^ Our study was carried out under controlled diet and with collection of urine samples before and after coffee intervention. Because data on the contribution of coffee to the daily niacin intake are rather scarce, we performed a human intervention study with ten volunteers who each consumed 500 mL of freshly brewed coffee.

## 2 Experimental Section

### 2.1 Materials

Nicotinic acid, d_4_‐nicotinic acid (d_4_‐NA), nicotinamide, d_4_‐nicotinamide (d_4_‐NAM), *N* ^1^‐methylnicotinamide (NMNAM) iodide, d_3_‐*N* ^1^‐methylnicotinamide (d_3_‐NMNAM) iodide, *N* ^1^‐methyl‐2‐pyridone‐5‐carboxamide (2‐Py), and d_3_‐*N* ^1^‐methyl‐2‐pyridone‐5‐carboxamide (d_3_‐2‐Py) were obtained from Toronto Research Chemicals (Toronto, Canada). Acetonitrile was HPLC gradient grade and was acquired from LGC‐Standards (Wesel, Germany). Formic acid p.a. was purchased from Sigma‐Aldrich (Taufkirchen, Germany).

### 2.2 Preparation of the Coffee Beverage

Freshly brewed coffee was prepared using four coffee pads, each weighing 7.5 g (provided by Tchibo GmbH), giving a total of 30 g coffee powder. It was a medium dark roast coffee blend, made from mostly dark roasted and lightly roasted *Arabica* coffee, containing 11.38 mg g^−1^ caffeine. Each pad was extracted with 125 mL of hot water using a SENSEO coffee pad machine (Phillips, Amsterdam, Netherlands).

### 2.3 Study Design

**Figure 1:**
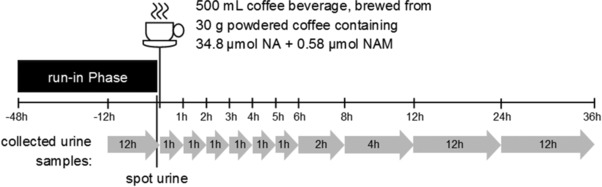
Design of the 4‐day human intervention study. Urine collecting periods are shown as grey arrows.

The human intervention study was performed with ten healthy, nonsmoking volunteers of Caucasian origin (six female and four male; age 22–27 years, BMI 22.6 ± 2.6 kg m^−2^), who habitually consume coffee on a daily basis. Exclusion criteria were age <20 or >50 years, BMI <19 or >25 kg m^−2^, smoking, being under medication and/or taking dietary supplements, being pregnant, performing competitive sport, not being accustomed to coffee consumption, not tolerating a 48 h abstinence from coffee, participating in other studies, or being a regular blood donor. All volunteers gave written consent to the study before inclusion, which was approved, No. 837.427.15(10195), by the ethical commission of the Rhineland‐Palatinate medical commission (Mainz, Germany). The study was performed in accordance with the ethical standards of the Declaration of Helsinki (1964), and lasted 4 d, during which the volunteers consumed standardized meals providing a low niacin intake. Detailed descriptions of meals are presented in Supporting Information 1. The first 2 d were run‐in days. During the last 12 h of this run‐in period, urine was collected overnight. In addition, spot urine samples were collected from each participant in the morning, immediately before coffee consumption. After the fast consumption of 500 mL coffee, total urine samples were collected during the following time periods: 0–1 h, 1–2 h, 2–3 h, 3–4 h, 4–5 h, 5–6 h, 6–8 h, 8–12 h, 12–24 h, 24–36 h (see **Figure** 1). Urine was weighed to determine the mass of urine produced by each participant, and aliquots were kept frozen at −20 °C until analysis. Total urine weight was converted into urine volumes assuming a density of 1 kg L^−1^.

### 2.4 Sample Preparation

Stock solutions of standards at concentrations of 1 mg mL^−1^ (NA, d_4_‐NA, NAM, d_4_‐NAM, NUA, d_4_‐NUA, NMNAM, d_3_‐NMNAM, 2‐Py, and d_3_‐2‐Py) were made in 0.1% aqueous formic acid and stored at −20 °C. These stock solutions were used to prepare diluted internal standard working solutions containing 2.5 μg mL^−1^ d_4_‐NA, 1.5 μg mL^−1^ d_4_‐NAM, 2.5 μg mL^−1^ d_4_‐NUA, 17.5 μg mL^−1^ d_3_‐NMNAM, and 50 μg mL^−1^ d_3_‐2‐Py.

All analytes were determined in triplicates. The internal standard solution (15 μL) was added to a urine sample aliquot (735 μL). After equilibration, 750 μL of acetonitrile (−20 °C) was added for protein precipitation and the sample was shaken for 5 min at 10 °C and 800 rpm (Thermomixer comfort, Eppendorf, Hamburg, Germany). Protein was then removed by centrifugation (13 000 rpm, 4 °C, 10 min) and the supernatant was transferred to a micro reaction tube. The sample was concentrated to a volume of 100 μL using a vacuum concentrator (Concentrator plus, Eppendorf, Hamburg, Germany) and then diluted to a total volume of 750 μL with 0.1% aqueous formic acid. Aliquots of 5 μL were analyzed by the HPLC‐MS/MS method described below.

### 2.5 HPLC‐ESI‐MS/MS Analysis of Urinary Metabolites

The HPLC system (1200 series, Agilent, Waldbronn, Germany) was coupled to an API 3200 triple quadrupole mass spectrometer (Applied Biosystems, Darmstadt, Germany) with ESI‐source. NA, NAM, and their metabolites were separated with a Synergi 4u polar‐RP column (250 × 4.6 mm, Phenomenex, Torrance, USA), equipped with the appropriate guard column at 20 °C using 0.1% aqueous formic acid (A) and acetonitrile (B) at a flow rate of 800 μL min^−1^. Gradient started at 0.5% B, isocratic for 2 min, then B was raised to 12.5% over 4 min. From 6.1 to 8 min, B was increased from 32.5% to 40% and then kept isocratic for an additional 2 min. Thereafter, the column was rinsed with 80% B for 7 min, to prepare for the next cycle, which was started after at least 7 min equilibration under the initial solvent conditions. The ESI‐source was operated in positive ion mode (2,500 V), and nitrogen was used as nebulizer gas (60 psi), heater gas (60 psi, 400 °C), curtain gas (30 psi), and collision gas (2 psi). Niacin and its metabolites as well as the isotopically labelled standards were detected in multiple reaction monitoring (MRM) mode. MS parameters and chromatograms are displayed in Supporting Information 2.

### 2.6 Quantitative Analysis and Method Validation

Standard solutions for calibration curve were prepared from the stock solutions described above in concentrations from 10 to 200 ng mL^−1^ for NA and NAM, 10 to 1000 ng mL^−1^ for NMNAM, and 10 to 3000 ng mL^−1^ for 2‐Py. Each standard solution contained 15 μL mL^−1^ of internal standard working solution, to obtain the same concentrations as in samples. For calibration, the observed peak area ratios were plotted against the concentration ratios, yielding a correlation coefficient of R^2^ > 0.99. Signal‐to‐noise ratios were defined as 1:3 relative to the LOD and 1:10 relative to the LOQ, listed in Supporting Information 2.1. The precision of the HPLC‐MS/MS‐method was determined by repeatedly measuring a standard mixture containing 100 ng mL^−1^ of each standard. The intra‐day coefficient of variation was ≤3.1%, and the inter‐day coefficient of variation based on analyses performed on 5 consecutive days was ≤ 5.6%. Recoveries were determined by spiking urine with 80%, 100%, and 120% of the expected amounts of NA, NAM, NMNAM, and 2‐Py. They were 76 ± 4% for NA, 119 ± 31% for NAM, 113 ± 13% for NMNAM, and 87 ± 9% for 2‐Py, respectively.

### 2.7 Analysis of the Coffee Beverage

Aliquots (*n* = 3) of the coffee beverage were diluted with 0.1% aqueous formic acid 1:100 and 1:200 for NA detection, and 1:20 and 1:50 for analysis of NAM. The diluted coffee samples were spiked with 100 ng mL^−1^ of the corresponding isotopically labelled standard. After equilibration, the samples were passed through a syringe filter (Chromafil AO‐45/25, polyamid, 0.45 μm, Macherey‐Nagel, Düren, Germany) and a 5 μL aliquot was analyzed using the above‐described method for urinary metabolites.

### 2.8 Data Analysis

Data were processed by Analyst 1.6 and Multiquant 2.0 (AB Sciex, Darmstadt, Germany). Experimental results are reported as means of at least three independent preparations and analyses ± SD. Statistical evaluations (Anderson–Darling test and two‐sample *t*‐test) were performed with Origin 2016 (OriginLab, Northampton, USA); differences were considered significant based on *p*‐values of *p* ≤ 0.05, *p* ≤ 0.01, and *p* ≤ 0.001. To determine the influence of coffee consumption, baseline excretion levels of each niacin compound and metabolite were defined by averaging the levels observed in urine samples collected during the overnight period immediately before (−12 to 0 h) and 12 h after coffee consumption (12–24 h).

## 3 Results

We performed a 4‐day human intervention study with four male and six female volunteers. For 48 h prior to start and during the 2 study days volunteers ingested a low‐niacin diet, and urine samples were collected regularly. After 48 h, on day 3, they consumed a bolus of 500 mL of coffee containing 34.8 μmol NA and 0.58 μmol NAM, respectively, analyzed by our established HPLC‐ESI‐MS/MS method.

**Figure 2:**
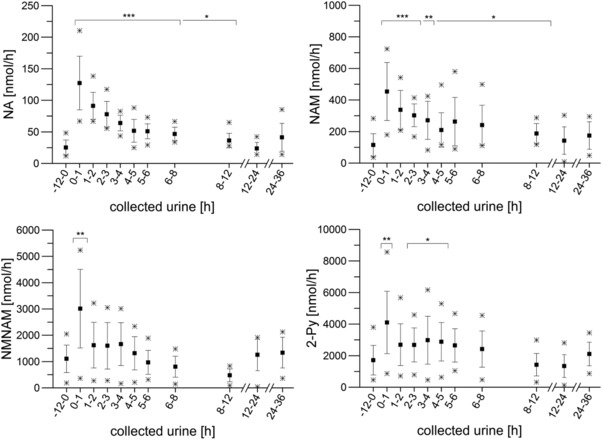
Excretion kinetics for nicotinic acid (NA), nicotinamide (NAM), *N* ^1^‐methyl‐nicotinamide (NMNAM), and *N* ^1^‐methyl‐2‐pyridon‐5‐carboxamide (2‐Py) based on urine samples collected before (−12 to 0 h) and after coffee intake (0–1 h, 1–2 h, 2–3 h, 3–4 h, 4–5 h, 5–6 h, 6–8 h, 8–12 h, 12–24 h, 24–36 h). Shown are the mean values (*n* = 10) with standard deviation as well as the minimum and maximum excretion rate. Statistical significance of excretion versus excretion before consumption of coffee is marked with *** for *p* ≤ 0.001, ** for *p* ≤ 0.01, and * for *p* ≤ 0.05.

**Figure 3:**
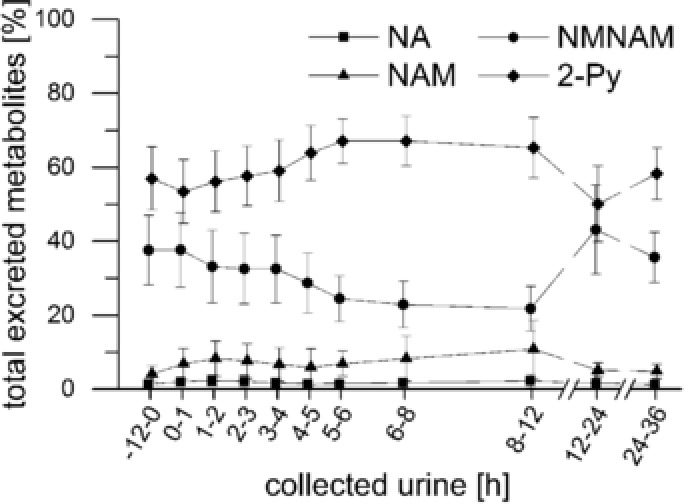
Relative distribution of nicotinic acid (NA), nicotinamide (NAM), *N* ^1^‐methyl‐nicotinamide (NMNAM), and *N* ^1^‐methyl‐2‐pyridon‐5‐carboxamide (2‐Py) in urine samples of all analyzed metabolites.

NA, NAM, and their metabolites in coffee and urine samples were identified by cochromatography in MRM‐mode with reference compounds. In addition to NA and NAM, the metabolites NMNAM, 2‐Py, and NUA were quantified by SIVA using HPLC‐ESI‐MS/MS. All the metabolites were present in the urine collected during the run‐in period, but their levels increased significantly (*p* ≤ 0.01 for NMNAM, 2‐Py; *p* ≤ 0.001 for NA, NAM) shortly after coffee consumption. Traces of NUA (<LOQ) were barely detectable shortly after coffee intake (0–1 h) in urine samples of some participants, whereas the other metabolites (NA, NAM, NMNAM, 2‐Py) reached their *t*_max_ (NA: 127.4 ± 42.5 nmol h^−1^; NAM: 453 ± 184 nmol h^−1^; NMNAM: 3014 ± 1494 nmol h^−1^; 2‐Py: 4102 ± 1976 nmol h^−1^). The baseline excretion was 25.2 ± 11.8 nmol h^−1^ for NA, 129 ± 77 nmol h^−1^ for NAM, 1180 ± 519 nmol h^−1^ for NMNAM, and 1527 ± 814 nmol h^−1^ for 2‐Py, respectively. After 12 h, the levels of all analyzed compounds were back to baseline concentrations. In total an extra 972 ± 966 nmol of all compounds was excreted during the first 12 h after coffee intake (relative to the baseline). Elimination of each analyte over the first 12 h after coffee intake was 34.1 ± 7.3 nmol h^−1^ for NA, 127 ± 58 nmol h^−1^ for NAM, −38 ± 523 nmol h^−1^ for NMNAM, and 849 ± 485 nmol h^−1^ for 2‐Py. The excretion kinetics of NA, NAM, NMNAM, and 2‐Py are shown in **Figure** 2. Niacin metabolites were excreted at an average rate of 972 ± 966 nmol h^−1^ during the first 12 h after coffee consumption, corresponding to an increase of 21% relative to 24–36 h‐period (804 ± 1294 nmol h^−1^). The excretion of NA on day 3 (coffee intake) represents 202% of the excretion on day 4, when no coffee was consumed (278% for NAM, 24% for NMNAM, and 146% for 2‐Py). In total, 8 ± 8% and 9 ± 14% of the ingested NA and NAM, respectively, was excreted on day 3 (0–12 h) and day 4 (24–36 h). The differences in excreted metabolites from day 3 to day 4 represent 6% of the niacin, which was supplied with coffee. The percent distribution of the metabolites in the excreted urine were 1.5 ± 1.5% for NA, 6 ± 3% for NAM, 36 ± 9% for NMNAM, and 57 ± 8% for 2‐Py. Changes in the distribution of all four analyzed metabolites (NA + NAM + NMNAM + 2‐Py = 100%) over time are shown in **Figure** 3.

## 4 Discussion

We performed a controlled human intervention study with a 48 h run‐in‐phase during which the participants consumed a low niacin diet. All ten participants consumed this diet for four days. After the run‐in, the participants consumed 500 mL coffee, and their urine was collected at hourly intervals to determine kinetic parameters such as *t*_max_ and *v*_max_. The results provide detailed quantitative data on the excretion kinetics of NA and NAM metabolites after coffee consumption. In addition to natural niacin content in foods, the endogenous formation from tryptophan, as well as the degradation of the coenzymes NAD and NADP contribute to the background excretion of NA, NAM, and its metabolites. The urinary excretion of all metabolites increased significantly after coffee consumption reaching peak levels within the first hour after coffee consumption. The levels of all studied metabolites returned to their baseline values within 12 h after coffee consumption, albeit participants showed large interindividual differences in renal excretion. Besides dietary factors, other life‐related effects, such as physical activity, may have led to this interindividual variation, but were not controlled in the frame of this study.

EFSA^1^ data suggest a population reference intake for niacin of 1.6 mg NE MJ^−1^ (6.6 mg per 1000 kcal). In our study, the intake of NA and NAM due to food (without coffee) was 14.1 mg NE d^−1^ (day 1 + 3, 2,365 kcal) and 13.6 mg NE d^−1^ (day 2 + 4, 2,302 kcal) summing up for 90% of the recommended daily niacin intake. In the intervention, the participants drank 500 mL coffee containing 4.36 mg NE. This one shot uptake was chosen deliberately to ascertain detectability of intake‐associated variations in urinary exposure biomarker kinetics. It may not reflect exactly the normal situation of regular coffee consumption, which may rather occur in several smaller portions during the day and would be expected to lead to more sustained levels of urinary niacin metabolites. Future investigations may address the influence of coffee intake, as consumed by typical coffee drinkers in more detail.

The relative abundancies of 2‐Py (57%) and NMNAM (36%) observed in this work are at the upper limits of the ranges reported in the literature (45–60% for 2‐Py and 20–35% for NMNAM).^1^, ^14^, ^15^

The excretion of NA, NAM, and its metabolites within 12 h after coffee consumption was found to account for 6% of the niacin ingested with coffee. Higher intakes at pharmacological doses were found to result in a noticeably higher excretion of niacin metabolites, varying between 40% and 75%.^23^, ^24^, ^25^ In our study, the diet‐related low single‐dose intake of niacin with coffee resulted in lower recovery of renal metabolites. Using a low niacin diet, the present study allowed to measure the influence of coffee intake on the urinary excretion of niacin metabolites. The rapid increase of urinary excretion directly after coffee consumption reflects the fast absorption of niacin. Again, limiting factors of our study were the bolus intake of 500 mL of coffee here and the fact that other lifestyle factors might have influenced the excretion of niacin metabolites from tryptophan and NAD/NADP turnover. Altogether, the results allow to conclude that coffee consumption on a regular basis notably contributes to niacin intake in humans.

**Abbreviations**
2‐Py: *N* ^1^‐methyl‐2‐pyridone‐5‐carboxamide
NA: nicotinic acid
NAM: nicotinamide
NE: niacin equivalent
NMNAM: *N* ^1^‐methylnicotinamide
NUA: nicotinuric acid
SIVA: stable isotope dilution analysis

## Conflict of Interest

The authors declare no conflicts of interest. G. Eisenbrand is a scientific advisor of Tchibo GmbH.

## Supporting information

Supporting Information
