# Supplementary material for: Urinary Excretion of Niacin Metabolites in Humans After Coffee Consumption
Source: Mol Nutr Food Res. 2018 Mar 23;62(7):1700735. doi: 10.1002/mnfr.201700735 (PMC5900739; doi:10.1002/mnfr.201700735)
Supplement: Supplementary file 1 — Supporting Information [file MNFR-62-na-s001.docx]

# Supplementary material

## Diet of human intervention study

The meals’ niacin content was calculated using data from the Bundeslebensmittelschluessel III, a food nutrition database maintained at the Max Rubner-Institut, Karlsruhe, Germany. [1] Each participant consumed about 2300 kcal per day, divided across four meals. The breakfasts provided on each day of the study consisted of a fruit salad containing peeled apples, pears and oranges (100 g each), with 200 g of creamy yoghurt (10 % fat) and 20 g of honey. On days one and three, the participants consumed 300 g noodles (containing eggs) with a cream sauce containing 100 g cream, 25 g fresh onions, and basil for lunch, followed by 200 g rice pudding and 150 g red fruit jelly as snack. They were also given a dinner consisting of 200 g boiled potatoes, 150 g carrots, and 100 g pumpkin with 50 g herb butter, followed by 125 g fruit jelly as dessert, flavored with raspberry or woodruff aroma. On days two and four, the lunch consisted of 250 g cooked (non-parboiled) rice, 50 g herb butter and 300 g of cooked vegetables (carrot, aubergine, and zucchini, in a 1:1:1 ratio). On these days, the snack was 200 g lemon sorbet, 125 g raspberries, and 40 g white chocolate. A mixed salad cup, containing 50 g iceberg lettuce, 75 g yellow peppers, 75 g cucumber and 75 g tomatoes was served with a soup prepared of 350 g tomatoes, 25 g onions, 75 g boiled pasta and 50 g sour cream as dinner completed with 125 g jelly. The participants drank water ad libitum.

## HPLC-ESI-MS/MS analysis urinary metabolites

### MS parameters and retention times

Table S2.1: Ion transitions, retention times and compound-specific MS parameters for nicotinic acid (NA), d_4_‑NA, nicotinamide (NAM), d_4_‑NAM, nicotinuric acid (NUA), d_4_‑NUA, *N*^1^‑methylnicotinamide (NMNAM), d_3_‑NMNAM, *N*^1^‑methyl-2‑pyridone‑5‑carboxamide (2-Py), d_3_‑2-Py (Agilent 1200 HPLC + API 3200 MS). Mass transitions labelled with * were used for quantification. DP = declustering potential, EP = entrance potential, CEP = cell entrance potential, CE = collision energy, CXP = cell exit potential. Limit of detection (LOD) and Limit of quantification (LOQ) of the analyzed compounds (NA, NAM, NUA, NMNAM, 2‑Py) in fmol. Signal to noise ratios were defined as 1:3 respectively 1:10 (cf. materials and methods section)

| compound | ion transitions [m/z] | retention time [min] | LOD [fmol] | LOQ [fmol] | DP  [V] | EP  [V] | CEP [V] | CE  [V] | CXP [V] |
| --- | --- | --- | --- | --- | --- | --- | --- | --- | --- |
| NA | 124.0 / 80.2* | 7.1 | 81 | 406 | 56 | 12 | 10 | 29 | 4 |
|  | 124.0 / 78.0 |  |  |  | 56 | 12 | 10 | 29 | 4 |
| d_4_-NA | 128.0 / 84.1* |  |  |  | 56 | 11.5 | 10 | 27 | 4 |
| NAM | 123.0 / 79.9* | 8.4 | 82 | 409 | 61 | 9 | 10 | 25 | 4 |
|  | 123.0 / 77.8 |  |  |  | 61 | 9 | 10 | 31 | 4 |
| d_4_-NAM | 127.0 / 84.2* |  |  |  | 46 | 10.5 | 12 | 31 | 4 |
| NUA | 181.2 / 135.1* | 9.7 | 278 | 1110 | 51 | 9 | 14 | 23 | 4 |
|  | 181.2 / 79.1 |  |  |  | 51 | 9 | 14 | 55 | 4 |
| d_4_-NUA | 185.2 / 139.0* |  |  |  | 51 | 9 | 14 | 23 | 4 |
| NMNAM | 137.2 / 94.1* | 5.9 | 36 | 146 | 61 | 10 | 12 | 27 | 4 |
|  | 137.2 / 92.0 |  |  |  | 61 | 10 | 12 | 27 | 4 |
| d_3_-NMNAM | 140.1 / 97.1* |  |  |  | 56 | 10 | 12 | 27 | 4 |
| 2-Py | 153.2 / 108.0* | 14.2 | 81 | 406 | 56 | 10 | 12 | 29 | 4 |
|  | 153.2 / 110.0 |  |  |  | 56 | 10 | 12 | 29 | 4 |
| d_3_-2-Py | 156.2 / 111.2* |  |  |  | 56 | 10 | 10 | 29 | 4 |

### Representative HPLC-ESI-MS/MS chromatograms

Figure S2.2.1: HPLC-ESI-MS/MS chromatogram of NA (Rt = 7.1 min) in the MRM mode (*m/z* 124.0 / 80.2) of a representative urine sample.

Figure S2.2.2: HPLC-ESI-MS/MS chromatogram of d_4_-NA (Rt = 7.1 min) in the MRM mode (*m/z* 128.0 / 84.1) of a representative spiked urine sample.

Figure S2.2.3: HPLC-ESI-MS/MS chromatogram of NAM (Rt = 8.4 min) in the MRM mode (*m/z* 123.0 / 79.9) of a representative urine sample.

Figure S2.2.4: HPLC-ESI-MS/MS chromatogram of d_4_-NAM (Rt = 8.4 min) in the MRM mode (*m/z* 127.0 / 84.2) of a spiked urine sample.

Figure S2.2.5: HPLC-MS/MS chromatogram of NMNAM (Rt = 5.9 min) in the MRM mode (*m/z* 137.2 / 94.1) of a representative urine sample.

Figure S2.2.6: HPLC-ESI-MS/MS chromatogram of d_3_-NMNAM (Rt = 5.9 min) in the MRM mode (*m/z* 140.1 / 97.1) of a spiked urine sample.

Figure S2.2.7: HPLC-ESI-MS/MS chromatogram of 2-Py (Rt = 14.2 min) in the MRM mode (*m/z* 153.2 / 108.0) of a representative urine sample.

Figure S2.2.8: HPLC-MS/MS chromatogram of d_3_-2-Py (Rt = 14.2 min) in the MRM mode (*m/z* 156.2 / 111.2) of a spiked urine sample.

## Supplementary references

[1] Max Rubner-Institut, Bundesforschungsinstitut für Ernährung und Lebensmittel, *Bundeslebensmittelschlüssel III*, http://www.ernaehrung.de/lebensmittel/ (accessed August 1, 2017).
